# In vitro study of nanoliposomes containing curcumin and doxycycline for enhanced antimicrobial photodynamic therapy against *Aggregatibacter actinomycetemcomitans*

**DOI:** 10.1038/s41598-023-38812-4

**Published:** 2023-07-18

**Authors:** Shima Afrasiabi, Alireza Partoazar, Nasim Chiniforush

**Affiliations:** 1 Laser Research Center of Dentistry, Dentistry Research Institute, Tehran University of Medical Sciences, Tehran, Iran; 2 Experimental Medicine Research Center, Tehran University of Medical Sciences, Tehran, Iran

**Keywords:** Microbiology, Diseases, Health care, Medical research, Nanoscience and technology

## Abstract

The excessive inappropriate use of systemic antibiotics has contributed to the emergence of antibiotic-resistant pathogens, which pose a significant risk to the success of treatment. This study has approached this problem by developing doxycycline-loaded liposome doped with curcumin (NL-Cur^+Dox^) for combination antibacterial therapy against *Aggregatibacter actinomycetemcomitans*. The characterization of formulation revealed encapsulation of both drugs in NL-Cur^+Dox^ with an average size of 239 nm and sustained release behavior. Transmission electron microscopy analysis confirmed the vesicular-shaped nanocarriers without any aggregation or crystallization. The cytotoxic and hemolytic activities of NL-Cur^+Dox^ were evaluated. The anti-biofilm and anti-metabolic effects of NL-Cur^+Dox^ -mediated antimicrobial photodynamic therapy (aPDT) were examined. The data indicated that NL-Cur^+Dox^ -mediated aPDT led to a significant reduction of biofilm (82.7%, *p* = 0.003) and metabolic activity (75%, *p* < 0.001) of *A. actinomycetemcomitans* compared to the control. NL-Cur^+Dox^ had no significant cytotoxicity to human gingival fibroblast cells under selected conditions (*p* = 0.074). In addition, the hemolytic activity of NL-Cur^+Dox^ were negligible (< 5%). These findings demonstrate the potential application of such potent formulations in reducing one of the main bacteria causing periodontitis where the NL-Cur^+Dox^ could be exploited to achieve an improved phototherapeutic efficiency.

## Introduction

Periodontitis is defined by progressive degradation of periodontal supporting tissues, especially the alveolar bone loss. As the lesions progress, the alveolar bone height slowly decreases and the tooth base weakens. As a result, the roots of the teeth become exposed and the teeth become loose, eventually leading to tooth loss if left untreated^1^.

*Aggregatibacter actinomycetemcomitans* is a Gram-negative bacterium commonly isolated from the oral cavity of people with periodontal disease. Pro-inflammatory cytokines such as tumor necrosis factor (TNF)-α, interleukin-1 (IL-1), and IL-6 are increased in the presence of *A. actinomycetemcomitans* and cause osteoclast formation and bone resorption^2^. *A. actinomycetemcomitans* expresses components that can induce adhesion to mucosal surfaces, infiltrate epithelial cells, inhibit host defense mechanisms, and cause destruction of gingival tissue and alveolar bone loss^2,3^.

The success of periodontitis treatment depends on the elimination of the bacterial load from the periodontal pockets. Treatment of periodontal disease is challenging, and conventional, mechanical, or nonsurgical periodontal treatments cannot successfully reduce the microbial strain burden^4^. Unfortunately, many systemic antibiotic treatments are unable to suppress the bacteria that cause periodontal infections to undetectable levels^5^. However, treatment failure occurs, mainly because of significant side effects and the development of superinfection and bacterial resistance^6–8^. It has been observed that local route of drug delivery can have more than 100-fold concentration in the subgingival areas compared with systemic treatment, reducing the drug dose to the patient by 400-fold^8^. However, there are also limitations, for example, the drug dose is limited because the area is relatively small and high potency drugs are needed. For some products, there is a short-term duration in the pocket and is destroyed by the gingival crevicular fluid^9,10^.

Doxycycline (Dox) is a synthetic bacteriostatic tetracycline effective in periodontitis^11,12^. As a broad-spectrum antibiotic, Dox can affect the activity of periodontal pathogens. However, direct use of Dox, especially in the oral cavity, may lead to complications, such as systemic exposure and off-target toxicity, inadequate local administration, and poor response rate of this disease to systemic Dox. Therefore, Dox may be more effective in combination with a new drug carrier with a certain degree of targeting and slow release^11^. In the treatment of periodontitis, Dox should be administered several times daily to prevent insufficient concentration of the antibiotic in the subgingival areas. Therefore, the use of Dox with drug carriers is a more appropriate procedure of administering this antibiotic^13^.

Due to resistance limitations in the use of topical and systemic medications, antimicrobial photodynamic therapy (aPDT) has become popular in the control of oral and dental diseases. aPDT consists of three main components: a light source with a specific wavelength, a photosensitizer, and oxygen. After activation of the photosensitive substance and release of free radicals, the cell wall of pathogens is destroyed without causing microbial resistance^14,15^. It has been reported that the photosensitizers used in aPDT are very important for the efficiency of aPDT^16^.

Curcumin (Cur) is a natural photosensitizer with anti-inflammatory and antibacterial activities against various microorganisms^17^. Despite all benefits, the bioavailability of curcumin is poor due to low water solubility, rapid metabolism, rapid systemic excretion, low stability, insufficient tissue absorption, and sensitivity to physiological pH^17,18^. There does not appear to be a clinically relevant therapeutic effect of Cur^18^. Several studies have shown that the encapsulation efficiency of Cur in nanoparticles, including homogeneity and stability in solution, low polydispersity and zeta potential, increased anti-biofilm activity, improved photoinactivation efficiency of bacteria and reactive oxygen species (ROS) generation with negligible dark cytotoxicity^19–21^. On the other hand, the antibacterial effect of Cur is synergistic with several antibiotics and increases the susceptibility of bacteria to various antibiotics^22–24^. Bacterial resistance to various antibiotics is mediated by efflux transporters, and Cur possibly inhibits this efflux pump system^22^.

Nanocarriers were developed for sustained release of various types of molecules to overcome the possible drug overdose and the toxicity as well as increasing the solubility, stability, and drug efficiency^25,26^ Among the various nanocarriers, liposomes are biodegradable phospholipid vesicles with a bilayer membrane structure that have numerous advantages, including perfect biocompatibility, lack of toxicity, desirable interactions with target cells, ideal carriers for hydrophilic and/or hydrophobic compounds, and ease of preparation. This lipid-based carrier has shown promising results in the oral delivery of different therapeutic agents^26,27^. The carboxyl group in the liposome can attach to the amino group in the biofilm so that the liposome adheres to the biofilm. In addition, high drug loading ability and high stability under biological environment are other advantages of choosing liposomes as a nanocarriers^28^. Previous studies have shown that drug nanocarriers can penetrate the gingival groove^11^. Therefore, there is a demand to find new antimicrobial methods with minimum side effects.

Considering the benefits of Cur as well as Dox as an effective antibiotic for periodontitis, the present study was conducted to compare the effect of free Cur and Dox or a liposomal entrapped form during aPDT on *A. actinomycetemcomitans*.

## Results

### Characterization

**Figure 1:**
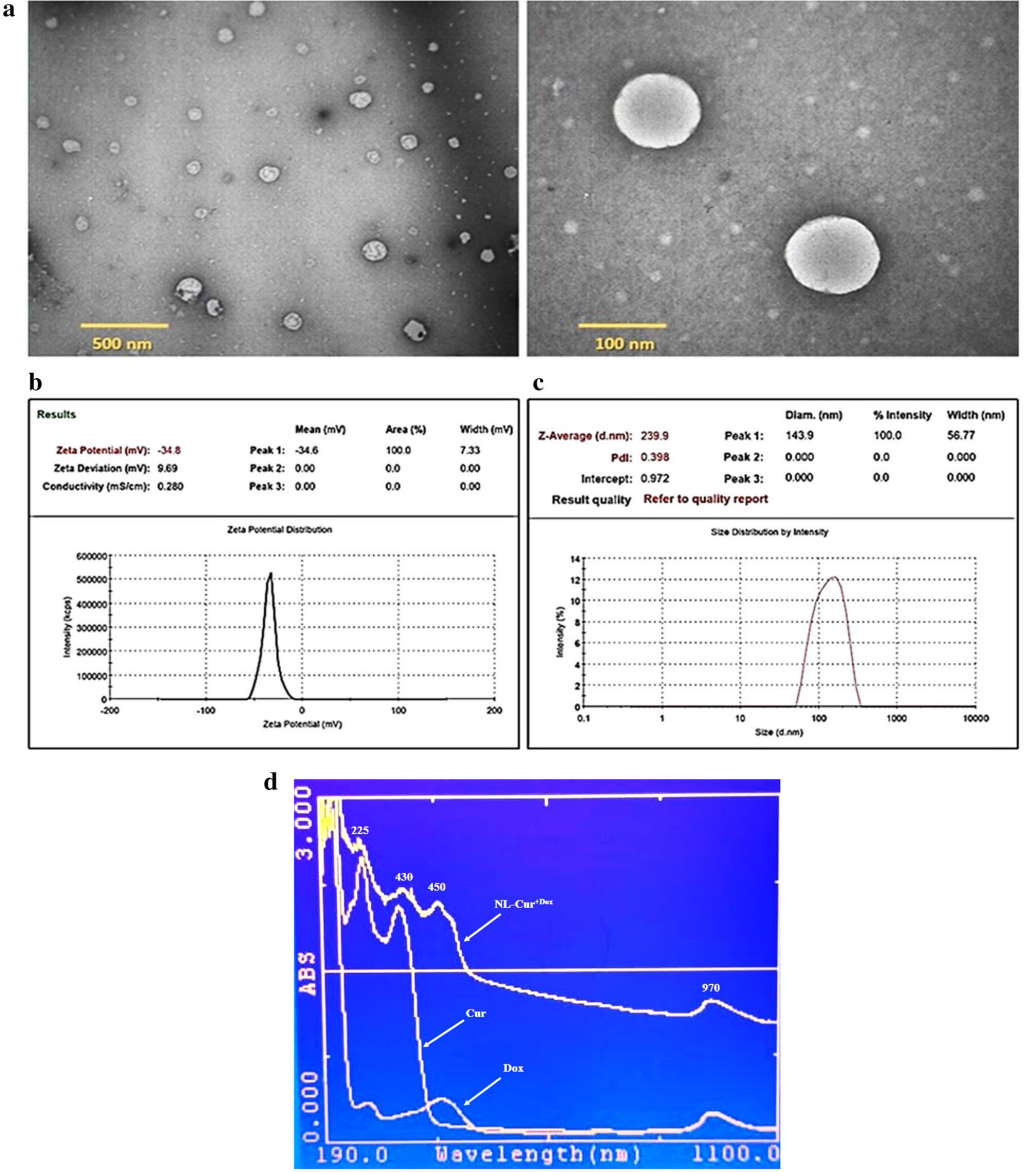
(**a**) Transmission electron microscopy image of NL-Cur^+Dox^ (**b**) Zeta potential of NL-Cur^+Dox^ (**c**) Dynamic light scattering of NL-Cur^+Dox^ (**d**) Absorption spectrum of Dox, Cur, and NL-Cur^+Dox^. Dox: Doxycycline, Cur: Curcumin, NL-Cur^+Dox^: Doxycycline-loaded liposome doped with curcumin.

In this study, we used the injection method to provide the spontaneous formation of liposomes concerning self-assembly of lipids into the liquid phase. It is well-recognized that active agents can be encapsulated inside the liposome core or in the lipid bilayer depending on their characteristics. Therefore, the lipophilic molecule like Cur should be integrated into the phospholipid bilayer and hydrophobic Dox is encapsulated in aqua core of the vesicular structure of NL-Cur^+Dox^. According to Table 1, this experiment determined an average size of 239 nm and surface charge of − 36 mV for NL-Cur^+Dox^ formulation. The encapsulation efficiency of both compounds was calculated approximately 47.2% and 83.4% for Dox and Cur, respectively. As shown in Fig. 1a, transmission electron microscopy (TEM) imaging of the nanoformulation displays the spherical shape of NLs without any aggregation or crystallization in the fields. Also, ζ-potential measurements and dynamic light scattering (DLS) analysis (Fig. 1b,c) shown negatively charged NLs which is related to phosphatidylserine phospholipids and a good polydispersity index (PDI) for NLs and is in correlation with TEM images in size of particles, respectively. The UV–Vis spectra of Dox, Cur, and NL-Cur^+Dox^ are presented in Fig. 1d.

**Table 1:** Physiochemical assessment of NL-Cur^+Dox^ formulation.

Test	Average size (nm)	PDI	Zeta potential (mV)	EE% (Cur)	EE% (Dox)
NL-Cur^+Dox^	239 ± 6.1	0.38 ± 0.08	− 36 ± 2.4	83.4 ± 2.3	47.2 ± 3.1

*NL-Cur*^+*Dox*^ doxycycline-loaded liposome doped with curcumin, *nm* nanometer, *PDI* polydispersity index, *mV* millivolt, *EE* encapsulation efficiency, *Cur* curcumin, *Dox* doxycycline.

### Drug release

**Figure 2:**
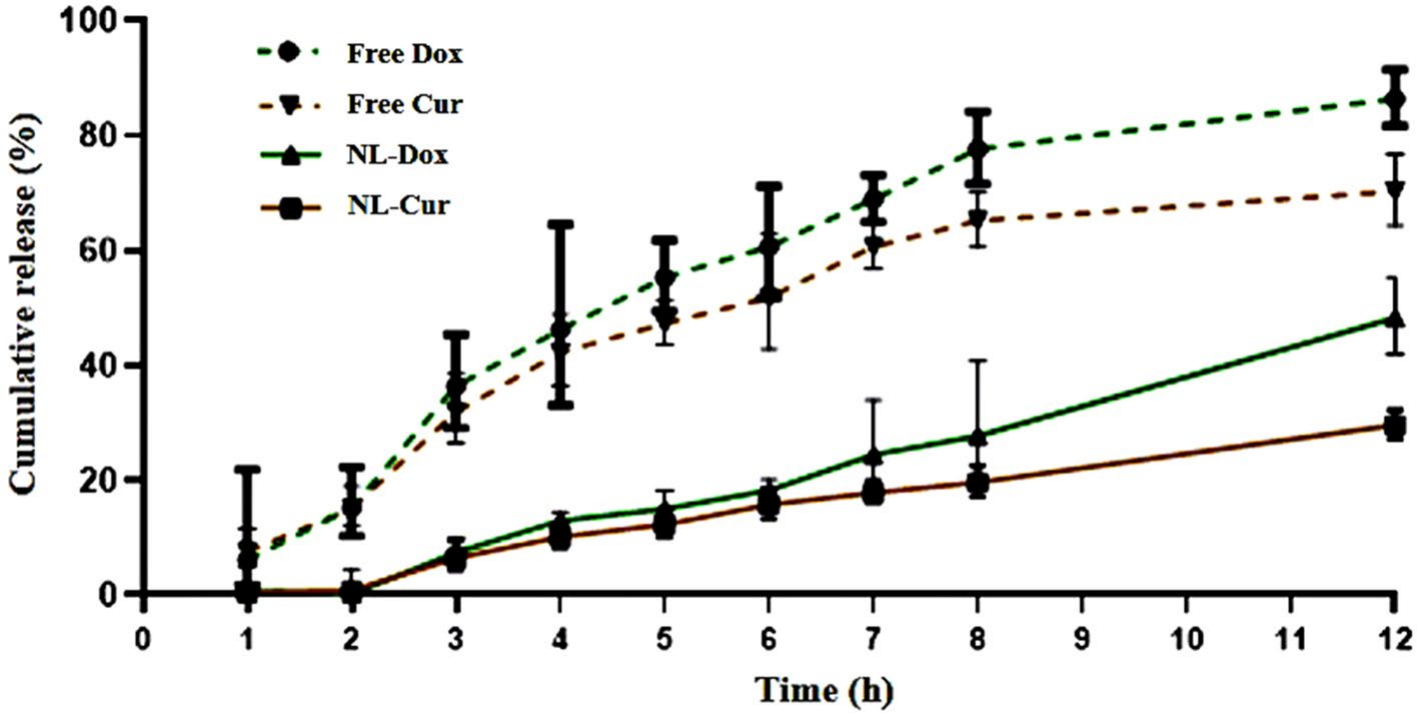
The representative release profile of doxycycline-loaded liposome doped with curcumin (NL-Cur^+Dox^) is compared to the free forms of Dox and Cur, respectively, in vitro conditions. *p = 0.0055 is versus free Dox. **p = 0.0012 is versus free Cur.

As shown in Fig. 2, data of the release profile determined a sustained release of the Dox and Cur molecules from the nanoformulation during 12 h in in vitro assessment. The release of hydrophilic Dox molecules from NL-Cur^+Dox^ occurred 18.6% more than lipophilic Cur molecules into the receptor media at the end of the experiment. The t-test analysis showed that Cur as NL-Cur^+Dox^ was released significantly, *p* = 0.0012, lesser than the free form of Cur into the media. The percentage of Cur release from formulation was 29.67 ± 1 into the receptor media while the release percentage was 70.37 ± 2.51 for free Cur after 12 h investigation. Moreover, the release number of formulated Dox, 48.33 ± 2.6%, was decreased significantly (*p* = 0.0055) in compression with free Dox with the number of 86.47 ± 1.95% at the 12 h time interval.

### MIC of Dox and Cur in NL form

As the MIC value reflects complete inhibition of growth of bacteria and the absence of production of virulence factors. Therefore, sub-MIC doses were selected for evaluating the effect of Dox and Cur in free or liposomal form on the modulation of biofilm formation. The MIC values of Dox and Cur in NL form were 7.8 and 78.1 μg/mL, respectively, whereas the sub-MIC values were 3.9 and 39.0 μg/mL Dox and Cur in NL form, respectively.

### Anti-bactericidal effect

**Figure 3:**
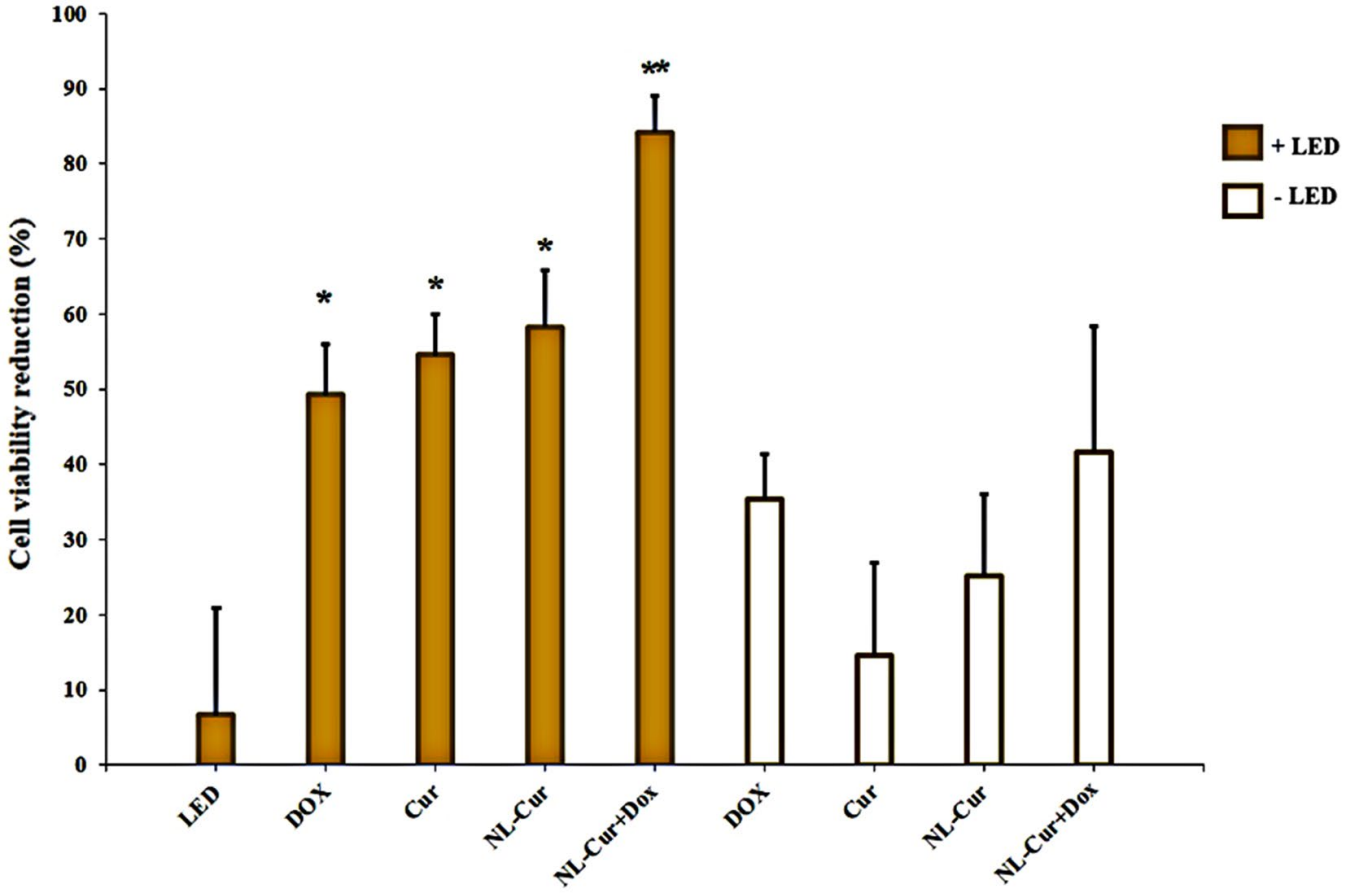
Cell viability reduction of *Aggregatibacter actinomycetemcomitans* in the experimental groups. *p* < 0.05 and < 0.001 are shown by * and **, respectively. Dox: Doxycycline, Cur: Curcumin, NL-Cur: Curcumin-loaded liposome, NL-Cur^+Dox^: Doxycycline-loaded liposome doped with curcumin, LED: Light-emitting diode.

The results of the efficacy tests of the experimental groups against *A. actinomycetemcomitans*, as determined by a CFU/mL assay, are shown in Fig. 3. The results show a significant reduction in *A. actinomycetemcomitans* bacterial counts by the ½ MIC dose of NL-Cur^+Dox^ with LED (84.2%, *p* < 0.001). Treatment with ½ MIC dose of Dox and Cur in free form and NL-Cur with LED irradiation resulted in a reduction of 49.2 (*p* = 0.029), 54.5% (*p* = 0.016), and 58.2% (*p* = 0.012), respectively. Moreover, in the absence of LED, ½ MIC doses of Dox, Cur, NL-Cur, and NL-Cur^+Dox^ reduced the number of *A. actinomycetemcomitans* to 35.4 (*p* = 0.27), 14.6 (*p* = 1.00), 25.25 (*p* = 0.901), and 41.6% (*p* = 0.159), respectively. In addition, LED irradiation for 60 s with a reduction of 6.7% did not show any significant difference from the control (*p* = 1.00). The results confirmed additive antimicrobial effects of NL-Cur^+Dox^ plus LED on *A. actinomycetemcomitans*.

### Anti-biofilm effects

**Figure 4:**
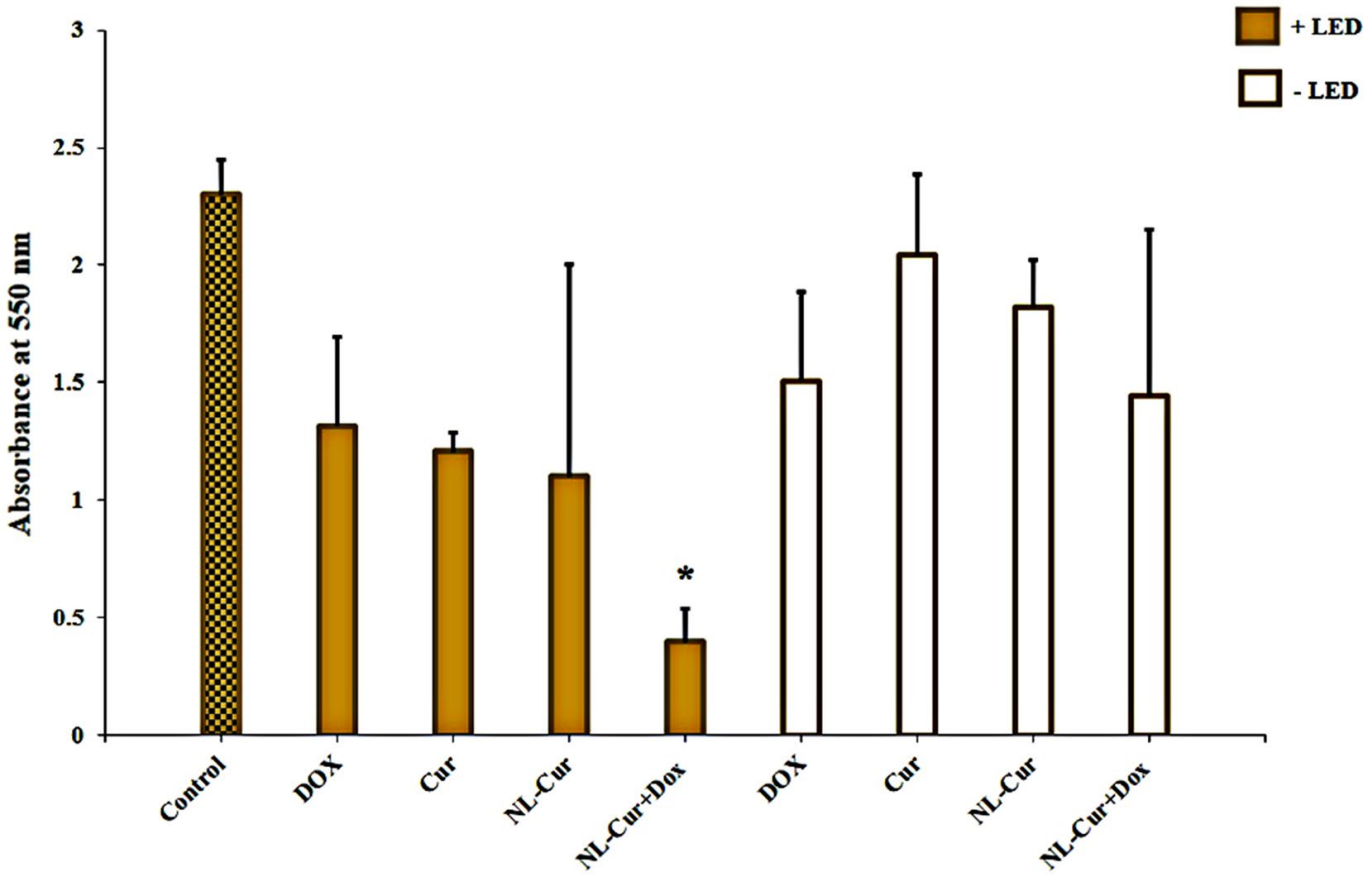
Biofilm reduction ability of *Aggregatibacter actinomycetemcomitans* in the experimental groups. *p* < 0.05 and < 0.001 are shown by * and **, respectively. Dox: Doxycycline, Cur: Curcumin, NL-Cur: Curcumin-loaded liposome, NL-Cur^+Dox^: Doxycycline-loaded liposome doped with curcumin, LED: Light-emitting diode.

As shown in Fig. 4, *A. actinomycetemcomitans* biofilms treated with Dox and Cur in the NL form at ½ MIC plus LED demonstrated superior biofilm reduction compared with other groups (82.7%, *p* = 0.003). Non-formulated Dox and Cur plus LED at the same concentration as NL form showed stronger anti-biofilm activity against *A. actinomycetemcomitans* biofilms than either group without LED irradiation, with reductions of 42.7 (*p* = 0.276) and 47.5% (*p* = 0.096), respectively. However, this reduction was not significant compared to the control. In addition, NL-Cur plus LED showed no significant change compared to control (52.1% reduction, *p* = 0.068). None of the groups without LED showed a significant difference from the control group.

### Anti-metabolic effect

**Figure 5:**
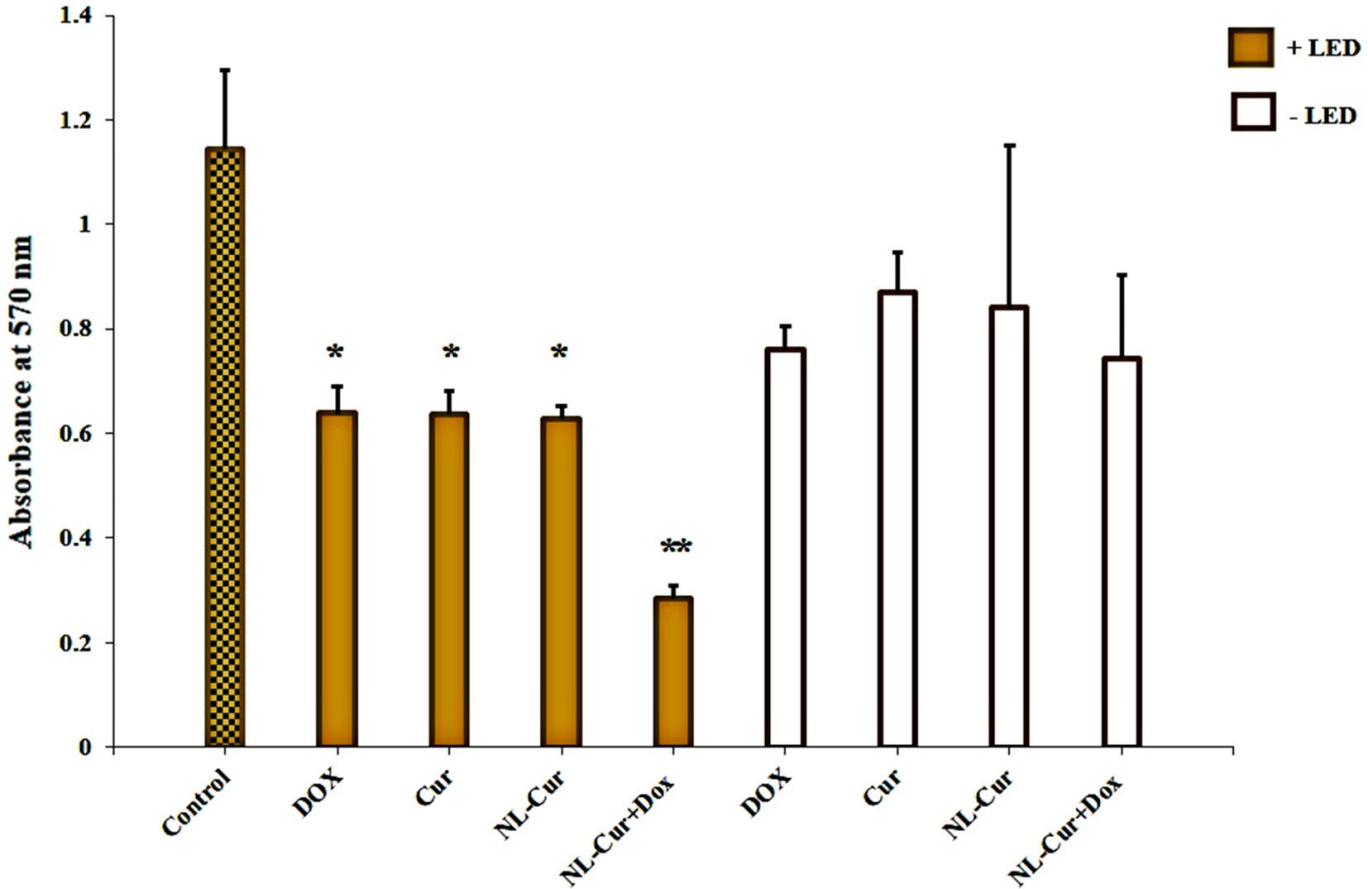
Metabolic activity reduction of *Aggregatibacter actinomycetemcomitans* in the experimental groups. *p* < 0.05 and < 0.001 are shown by * and **, respectively. Dox: Doxycycline, Cur: Curcumin, NL-Cur: Curcumin-loaded liposome, NL-Cur^+Dox^: Doxycycline-loaded liposome doped with curcumin, LED: Light-emitting diode.

Encapsulation of Dox and Cur in the formulations resulted in substantial attenuation of metabolic activity at ½ MIC, with 75% reduction (*p* < 0.001) after exposure to 3.9 μg/mL of Dox and 39.0 μg/mL of Cur in the NL form. However, non-formulated Dox, Cur, and NL-Cur plus LED caused a significant decrease in metabolic activity compared to other groups without LED (Fig. 5; *p* = 0.01, 0.004, and 0.003 respectively). There was also no significant change in the metabolic activity of *A. actinomycetemcomitans* following Dox, Cur, NL-Cur, and NL-Cur^+Dox^ without LED (*p* = 0.291, 0.359, 0.331, and 0.189, respectively).

### Cytotoxicity assay

**Figure 6:**
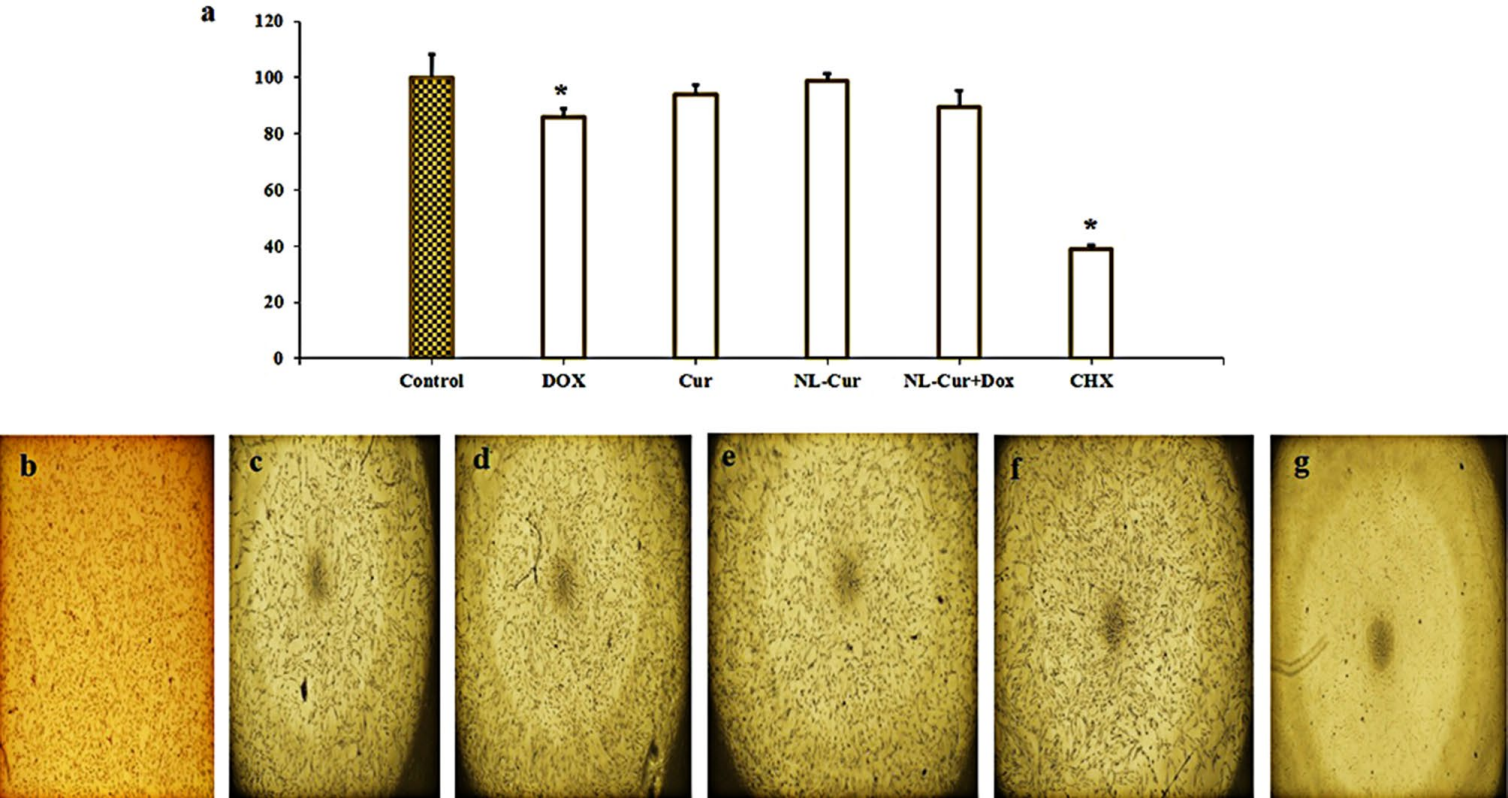
Cell cytotoxicity assessment. (**a**) MTT assay; photograph of HGF cells under inverted microscope (**b**) Control (**c**) Cells exposed to doxycycline (**d**) Cells exposed to Cur (**e**) Cells exposed to NL-Cur (**f**) Cells exposed to NL-Cur^+Dox^ (**g**) Cells exposed to CHX. *p* < 0.05 is shown by *. HGF: human gingival fibroblast, Dox: Doxycycline, Cur: Curcumin, NL-Cur: Curcumin-loaded liposome, NL-Cur^+Dox^: Doxycycline-loaded liposome doped with curcumin, CHX: Chlorhexidine.

The effect of Dox, Cur, NL-Cur, and NL-Cur^+Dox^ on human gingival fibroblasts (HGFs) was evaluated by cytotoxicity analysis using MTT assay (Fig. 6). Dox and Cur in free or liposomal form were used at ½ MIC concentrations and cell viability was assessed after 24 h of treatment. Cell viability data showed that unformulated Cur, NL-Cur, and NL-Cur^+Dox^ were biocompatible and maintained a high level of viability (up to 90%). However, cell viability changed significantly after treatment with free Dox (*p* = 0.007), indicating a significant effect on HGFs, implying that the antibiotic formulations in the NL maintain the safety. The high cytotoxicity was attributed to the 0.2% chlorhexidine (CHX) and cell viability reduced to 38.7% (*p* < 0.001).

### Hemolysis assay

**Figure 7:**
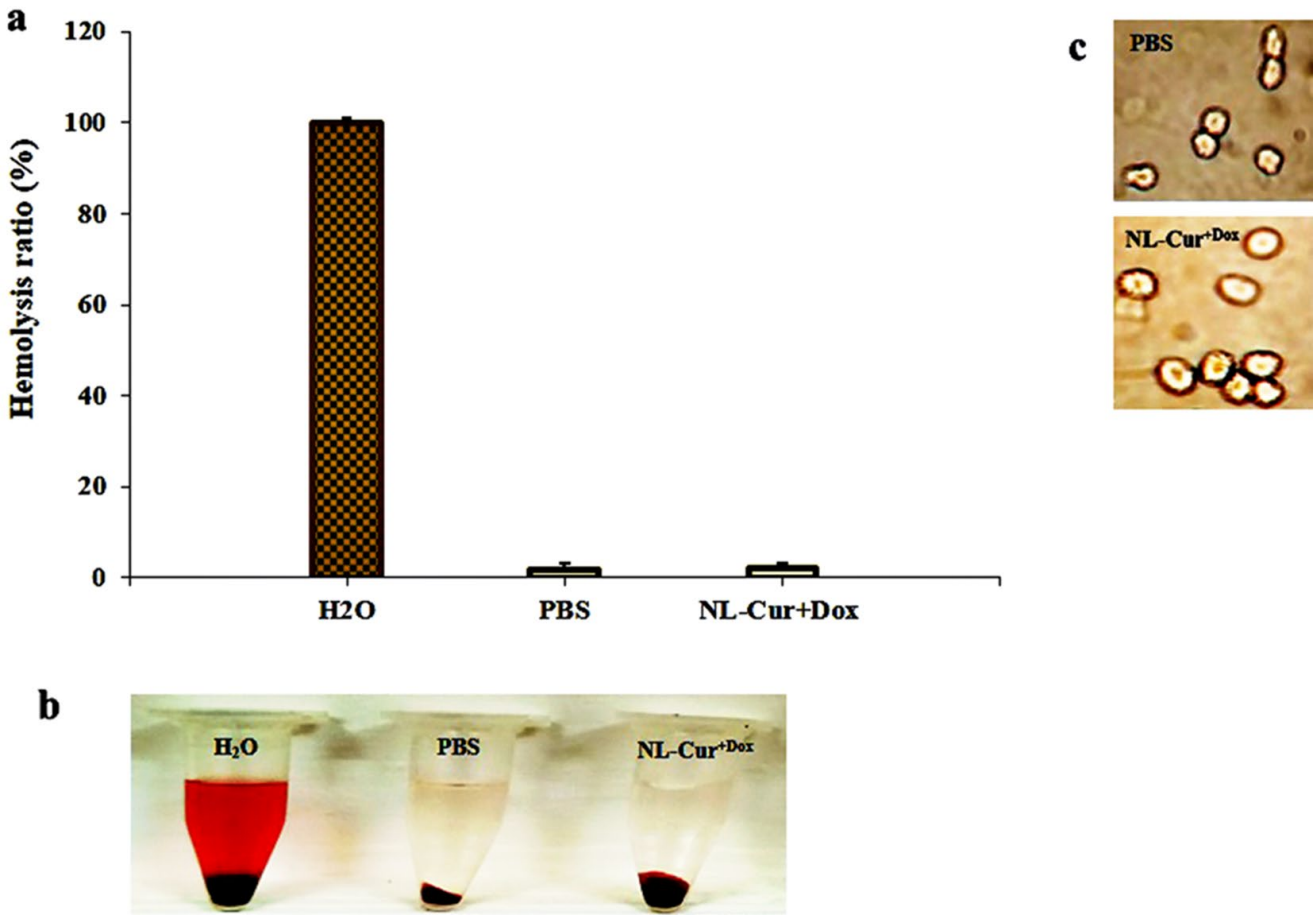
(**a**) Hemolysis ratio of RBCs treated with H_2_O (positive control), phosphate buffer saline (PBS, negative control), and NL-Cur^+Dox^ (**b**) Photographs of RBCs treated with H_2_O, PBS, and NL-Cur^+Dox^ (**c**) Optical images of RBCs treated with PBS, and NL-Cur^+Dox^. PBS: Phosphate buffer saline, NL-Cur^+Dox^: Doxycycline-loaded liposome doped with curcumin.

As shown in Fig. 7a, hemolysis ratios of RBCs were minimal for NL-Cur^+Dox^ at concentrations ½ MIC. Figure 7b shows the photographs of RBCs treated with NL-Cur^+Dox^ at ½ MIC concentration, similar to the negative control (phosphate buffer saline (PBS)). The RBCs treated with H_2_O show marked hemolysis. The corresponding optical images of RBCs showed that the RBCs had no morphological changes after incubation with NL-Cur^+Dox^ (Fig. 7c), further confirming the negligible hemolysis. All these results indicate that NL-Cur^+Dox^ has good hemocompatibility.

## Discussion

Biofilm formation is a critical virulence factor for *A. actinomycetemcomitans* involved in persistent periodontitis^29^. Microbial cells in a biofilm becomes more resistant to antimicrobial agents because the microorganisms are protected against the action of various agents such as antibiotics and host defenses^30^. The biofilm matrix also can adsorb the antimicrobial component and therefore reduce their bioavailability^31^. Moreover, microbial cells in biofilm structures show slow growth. In addition, conjugation between biofilm cells can play an important role in antimicrobial resistance^30^.

Overall, the results of the current study showed that the antibacterial and anti-biofilm activity of NL-Cur^+Dox^- mediated aPDT was apparently better than that of other groups. Unlike free Dox, the NL-Cur^+Dox^ itself showed no significant change on the viability of HGF cells. It also exhibited good hemocompatibility.

Here we selected negatively charged DPPS as well as DPPC phospholipids, resulting in more stability in harsh conditions and efficient drug delivery^32,33^. Our experimentation showed that both Dox and Cur could be encapsulated in the vesicles in the range of 47–83%, respectively. Interestingly, liposomes consist of the phospholipid bilayer to construct lipidic and aqueous phases that both hydrophobic and hydrophilic compounds can incorporate separately within compartments^34^. Therefore, photosensitive Cur^+Dox^ revealed that their synergy accelerates the anti-biofilm activity of the formulation in terms of aPDT against *A. actinomycetemcomitans*. Moreover, sustained release behavior of the formulation during 12 h examination will favor due to reduced cytotoxicity and enhanced bioavailability of the drugs^35^.

Sub-MIC doses of Dox are used as an adjunctive therapy for periodontitis because it decreases matrix metalloproteinases activity, which play a key role in promoting alveolar bone resorption and destruction of periodontal tissues. Dox also helps reduce connective tissue breakdown by decreasing the cytokine expression, and increasing bone formation, collagenolytic and osteoblast activity^36^. The susceptibility of some oral bacterial species to light irradiation in the presence of Dox has been previously demonstrated in laboratory research and in vivo experiments^37,38^. Conventional antibiotic-based strategies are no longer applicable mainly because of the problems of antibiotic resistance and the risks of abnormal side effects^39,40^. To reduce these problems, current treatment plans focus on the combined use of antibiotic with additional adjuvants^41^.

As previously reported, Cur-aPDT is an effective approach to reduce cell survival and virulence of *A. actinomycetemcomitans*^42,43^. Local application of Cur in slow-release vehicles resulted in positive antimicrobial and anti-inflammatory effects^44,45^. According to previous studies, the synergism between Cur antibacterial properties and the positive liposomes surface reduces its MIC against methicillin-resistant *Staphylococcus aureus* compared to its free forms^46,47^. There are previous studies reporting synergistic interactions between aPDT and various antibiotics^48–51^. aPDT with ROS production can inhibit the overexpression of active effector transporter proteins and reduces cellular activity. Meanwhile, chemotherapy can increase the sensitivity of bacterial cells to ROS^52^.

The use of NLs as a drug-delivery carrier in this context greatly improves the efficacy of antimicrobial agents and minimizes the recurrence of infections because of their unique properties, such as their flexibility in carrying both hydrophilic and hydrophobic agents, targeting ability, non-immunogenicity, and low toxicity^31,53,54^. One of the advantages of combination therapies is that there is less chance that the pathogen will resist multiple treatments simultaneously and a lower concentration of drugs is needed in combination than with the free form^49^. Therefore, in this study, a NL containing Dox and Cur plus LED was used to achieve a synergistic effect. Different studies have shown that the use of liposomal combination is more effective in eradicating different bacteria than the free form^55–57^.

Liposome-formulated drugs have already entered the clinics for the treatment of systemic or local infections. This is mainly due to the biocompatibility and the fact that they are biologically inert, and practically do not cause unwanted reactions^58^. One of the strengths of this study may be that it can pave the way for its clinical use. However, despite the advantages of these carriers, the main challenges in using NLs are their high sensitivity in response to temperature and pH changes, and premature release of hydrophilic bioactive compounds under long-term storage conditions. Therefore, much research should be done on the use of these compounds to stabilize and modify their structure and improve the preparation and storage methods to minimize the drawbacks. In addition, more studies should be conducted on the efficiency of these nanocarriers under in vivo situation^59^. These preliminary results shed light on the effects of aPDT using NL-Cur^+Dox^ on one of the most important bacteria involved in periodontitis. Although the use of one species is one of the limitations of this study. Another limitation of the study is that only ½ MIC are investigated. The NL-Cur^+Dox^- mediated aPDT should be investigated in future studies using different types of bacteria and multi-species bacterial biofilm models in periodontal research^60^. Moreover, the biofilm analysis using the xCelligence instrument is recommended^61^. The NL-Cur^+Dox^- mediated aPDT may also carry other drugs, and in vivo efficacy evidence is required to fully evaluate the potential of the compound.

## Conclusions

The nanoliposomal formulation provided co-encapsulation of Dox and Cur, which demonstrated sustained release behavior of the compounds in vitro assessment in this study. The slow release of NL-Cur^+Dox^ is a promising factor for reducing the toxicity of the drug as well as for long-lasting and local delivery. Our results suggest that aPDT induces high potential antimicrobial and anti-biofilm activity against *A. actinomycetemcomitans* through a synergy between Dox and Cur in the liposomal formulation. The biocompatibility of NL-Cur^+Dox^ in terms of hemocompatibility and lower HGF cytotoxicity are suitable indications for translation of the formulation to periodontitis medication. Moreover, the in vivo studies can stabilize our results and provide a better understanding of the mechanisms involved.

## Methods

### Preparation of NL-Cur^+Dox^

The preparation of NL-Cur^+Dox^ was conducted based on the injection method^62^. Briefly, the lipid mixture (total 400 mg) composed of 60% Dipalmitoylphosphathydilserine (DPPS), 30% dipalmitoylphosphathydilcholin (DPPC) (Avanti Polar Lipids, Alabaster, US), 10% Cholesterol (Merck, Darmstadt, Germany), and 5 mg/mL of Cur (UltraCur, weber medical, Germany) were dissolved at 40 °C. Then 0.5 mg/mL of Dox (Sigma-Aldrich, St. Louis, MO, US) was dissolved in 10 mL normal saline and added to the lipid mixture under a fine stream by a syringe while stirring at 700 rpm for 30 min. Finally, the suspension was homogenized for 5 min to obtain the NL-Cur^+Dox^ suspension. Meanwhile, NL-Cur without Dox was also prepared.

### Characterization

The mean diameter and surface charge of NL-Cur^+Dox^ were determined based on DLS by a Zetasizer system (Malvern Panalytical, Malvern, UK). NL morphology of the suspension was confirmed using TEM (Zeiss, Oberkochen, Germany). The percentage of encapsulation efficiency (EE %) was adapted from the procedures described earlier^41,63^. Briefly, Cur-Dox suspension was centrifuged at 8000×*g* (relative centrifugal force units) for 30 min at 4 °C. The supernatant was removed and then sediment was washed three times with saline. Then the recovered Cur of sediment (as dissolved completely in anhydrous methanol) and Dox of supernatant were measured by a UV spectrophotometer (Cecil, Cambridge, UK) at 430 nm and 271 nm, respectively. The percent EE% of both drugs in formulation was calculated by the following equation:

$${\text{EE }}\% \, = \, [({\text{total}}\,{\text{ amount }}\,{\text{of }}\,{\text{added}}\,{\text{ drug}}{ - }{\text{amount }}\,{\text{of}}\,{\text{ drug}}\,{\text{ in }}\,{\text{supernatant}}\,{\text{ or }}\,{\text{precipitant}}) \, /{\text{ total}}\,{\text{ amount}}\,{\text{ of }}\,{\text{added}}\,{\text{ drug}})] \, \times 100.$$

### In vitro drug release assessment

The release profile of NL-Cur^+Dox^ was evaluated by the in vitro Franz diffusion cell method^64^. Briefly, the 2 mL of suspension was placed between two chambers that were separated by a 12,000 Da cut-off dialysis membrane. The free drugs were released into the receptor phase, which was prepared as 70 mL PBS (pH: 7.4) containing 30% ethanol. It was achieved under a constant temperature of 37 °C and rotation speed of 100 rpm using heater stirrer. During 12 h, time intervals were determined and 0.5 mL of the sample was with-drawn from the receptor phase at each time. The receptor solution was subsequently replaced again with an equivalent volume of the sample. Then, the compounds’ concentration was measured using a UV spectrophotometer at the proper absorbance that were referred at the section of NL preparation. Also, equal concentration of free drugs was evaluated under the same condition.

### Bacterial strain and culture condition

*A. actinomycetemcomitans* ATCC 700,685 (HK1651, JP2 clone) was obtained from the American Type Culture Collection (Manassas, VA 20108 USA). It was grown on BHI broth (Merck, Darmstadt, Germany) with 5 mg/L of hemin (Sigma-Aldrich, St. Louis, MO, US), 1 mg/L of menadione (Sigma-Aldrich, St. Louis, MO, US), and 5 g/L of yeast extract (Ibresco, Iran) under 5% CO_2_ atmosphere at 37 °C.

### Light source

Blue LED (DentMate, New Taipei City, Taiwan) at wavelengths of 430–460 nm with output power density of 1000 ± 100 mW/cm^2^ and an energy density of 60 J/cm^2^ was used as a light source. The blue LED was placed at a distance of 1 mm away from the surface of the bacterial suspension. An irradiation dose of 60 s was considered^65^.

### Minimum inhibitory concentration (MIC)

Bacterial growth inhibition of formulations containing Dox and Cur was determined using the microbroth dilution technique described in the guideline of Clinical and Laboratory Standards Institute^66^. NL-Cur^+Dox^ was examined in two-fold dilutions in BHI broth in a Dox concentration range of 500–1 μg/mL, whereas Cur concentration range was 5000–10 μg/mL. One hundred mL of each material was placed in separate wells of a 96-well flat-bottomed plate (SPL Life Science, Pocheon, Gyeonggi, Korea). The wells were then inoculated with *A. actinomycetemcomitans* to yield a concentration of 1.5 × 10^5^ CFU/mL and incubated for 48 h at 37 °C in a 5% CO_2_ atmosphere.

### Study design

*A. actinomycetemcomitans* ATCC 700,685 was subjected to the following:

- Group 1: Control (without treatment)
- Group 2: Dox
- Group 3: Cur
- Group 4: NL-Cur
- Group 5: NL-Cur^+Dox^
- Group 6: Dox plus LED
- Group 7: Cur plus LED
- Group 8: NL-Cur plus LED

- Group 9: NL-Cur^+Dox^ plus LED

### Assessment of microbial viability

One hundred microliters of test materials (Dox, Cur, NL-Cur, and NL-Cur^+Dox^) at ½ MIC concentrations was separately added to the well of a 96-well flat-bottomed microplate. The wells were then inoculated with a 100 μL of fresh bacterial suspension (1.5 × 10^6^ CFU/mL). Five minutes were allowed for incubation at 25 °C. In groups 6–9, the microplate was exposed to the LED for 1 min. Then, a 10 μL aliquot of each well was serially diluted and cultured on a BHI agar (Merck, Darmstadt, Germany) plate at 37 °C with 5% CO_2_ atmosphere. The number of colonies was counted after 48 h.

### Assessment of anti‑biofilm activity

The biofilm of *A. actinomycetemcomitans* with initial inoculum of 1.5 × 10^6^ CFU/mL was formed in 96-well flat-bottomed microplate according to Haney et al.^67^. After treatment, the contents of each well were emptied, and samples were rinsed with PBS. The biofilms adhered to the bottom of the wells were fixed with methanol for 15 min, and stained with 0.1% crystal violet (Merck, Darmstadt, Germany) for 15 min. The remaining dye was removed with PBS. The microplate was air-dried and later the dye bound to the adhered bacteria was solubilized in 150 μL of 95% ethanol. The optical density of the plates was quantified by a microplate reader (Bio-Tek, Winooski, VT, USA) at 550 nm.

### Assessment of metabolic activity

The metabolic activity of *A. actinomycetemcomitans* was evaluated using the 3-(4,5-dimethylthiazol-2-yl)-2,5- diphenyltetrazolium bromide (MTT, Sigma-Aldrich, St. Louis, MO, US) assay, as described by Pandey et al.^68^. Following each treatment as explained in the experimental design section, MTT: PBS in the ratio of 1:10 was added to each of bacteria cultured sample, and incubated for 3 h at 37 °C. Reacting with the viable bacterial cells, MTT forms the formazan crystals which are dissolved using dimethylsulfoxide (DMSO, Sigma-Aldrich, St. Louis, MO, US) developing purple color. The absorbance of dissolved formazan crystals was measured at 570 nm wavelength, by microplate reader.

### Cell culture

HGFs (IBRC C10459) was purchased from Iranian Biological Resource Center (Tehran, Iran). HGFs were maintained in Dulbecco's modified Eagle's medium (DMEM, Biowest, Nuaillé, France) supplemented with 10% Fetal bovine serum (FBS, Gibco, Paisley, UK), 2 mM *L*-glutamine, and 1% pen/streptomycin (Biowest, Nuaillé, France). Cell cultures were kept in a humidified 5% CO_2_–95% air incubator at 37 °C. For cytotoxicity experiments, HGFs with 10,000 cells/well were seeded into 96-well plate. For cytotoxicity assays, cells were treated with Dox, Cur, NL-Cur, and NL-Cur^+Dox^ at ½ MIC concentrations for up to 24 h. Cells were then washed with PBS to remove non-adherent cells. Cytotoxicity was assessed using the MTT reduction assay. The absorbance was then measured at 570 nm using a microplate reader. In addition, the effect of CHX as a gold standard for periodontal disease on HGFs cells was investigated^69^. Cell morphology was assessed using an Olympus IX70 inverted microscope.

### Hemolysis assay

The following experimental method refers to Zhao et al.^70^. Blood was collected from a healthy adult male volunteer in Tehran, Iran. Human red blood cells (HRBCs) were placed in an anticoagulation tube and then treated with and NL-Cur^+Dox^ at ½ MIC concentrations or H_2_O (positive control) in PBS. RBCs in PBS was set as the negative control. Following incubation of the samples at 25 °C for 2 h, RBCs were centrifuged at 10,000 rpm for 1 min. The absorbance of supernatant was read using a microplate reader at 540 nm.

### Statistical analysis

The sample size was determined using One-Way Anova Power Analysis, PASS 11, considering the effect size = 0.75, *α* = 0.05, and *β* = 0.2, the minimum sample size required for each study group to measure the number of colonies is 6 samples and 3 samples are needed to measure biofilm. Data were analyzed using the statistical software package IBM SPSS Statistics version 26.0 (IBM, Chicago, IL, USA). The obtained data were statistically assessed by the one-way analysis of variance (ANOVA) followed Bonferroni test and *t*-test analysis was also used for drug release assessment. In order to check normality, Shapiro–Wilk test was used. The findings were expressed as mean ± standard deviation (SD), with *p* < 0.05.

### Ethics approval and consent to participate

Permission from the Ethics Committee of Tehran University of Medical Sciences was received before commencing the experiments (IR.TUMS.DENTISTRY.REC.1401.074), and all methods were carried out in accordance with relevant guidelines and regulations approved by the Tehran University of Medical Sciences (Protocol approval. no.: 1400–3-158–57230). All individuals who agreed to take part in the study signed informed consent forms prior to enrolment in this research.

## Data Availability

All datasets supporting the conclusions of this article are included within the article.
